# Complete remission of choriocarcinoma with pulmonary vein thrombosis in the third trimester of pregnancy treated with systemic chemotherapy and anticoagulation: A case report

**DOI:** 10.1097/MD.0000000000026145

**Published:** 2021-05-28

**Authors:** Xiaodong Li, Hongfa Peng

**Affiliations:** Department of Obstetrics and Gynecology, The Second Hospital of Hebei Medical University, Shijiazhuang, China.

**Keywords:** chemotherapy administration, choriocarcinoma, maternal and fetal safety, pregnancy care, pulmonary vein thrombus

## Abstract

**Rationale:**

Choriocarcinoma is a highly aggressive tumor. It occurs infrequently during pregnancy. The management of choriocarcinoma during pregnancy poses several challenges.

**Patient concerns:**

At 34 weeks of gestation, a 21-year-old primigravida was transferred to the emergency room for cephalgia, reduced fetal movements, and left intra-atrial intracavitary thrombus.

**Diagnosis:**

Choriocarcinoma in the third trimester with lung and brain metastases, pulmonary vein thrombosis (PVT), and systemic thrombosis

**Intervention:**

An emergency cesarean section was performed. Subsequently, low-molecular-weight heparin anticoagulation combined with multiagent chemotherapy was administered.

**Outcome:**

A 1.59 kg live female was born. Multiagent chemotherapy combined with anticoagulation led to complete regression of the cerebral and pulmonary lesions and the dissolution of pulmonary vein thrombus. At the 11-month follow-up, the patient remained in complete remission without complications, and her child was disease-free.

**Lessons:**

This is the first case of gestational choriocarcinoma with PVT. Our case suggests that conservative therapy can be the first choice for small, asymptomatic PVT secondary to choriocarcinoma.

## 1 Introduction

Choriocarcinoma is a rare and highly aggressive tumor.^[1]^ The diagnosis of choriocarcinoma during pregnancy is even rarer.^[2,3]^ Although uncommon, gestational choriocarcinoma can be fatal to both the mother and the fetus, and raises therapeutic, ethical, and social dilemmas for the treating physician.^[4–6]^ We encountered a rare case of choriocarcinoma with pulmonary venous thrombosis (PVT) and systemic thromboembolism in a patient in the third trimester of pregnancy. Complete remission was achieved with anticoagulation therapy combined with multiagent chemotherapy. We reviewed the related literature using a PubMed search and found 4 cases of gestational choriocarcinoma from 1966 till date. To the best of our knowledge, this is the first report of gestational choriocarcinoma with PVT that was successfully treated with anticoagulation combined with chemotherapy.

## 2 Case presentation

**Figure 1:**
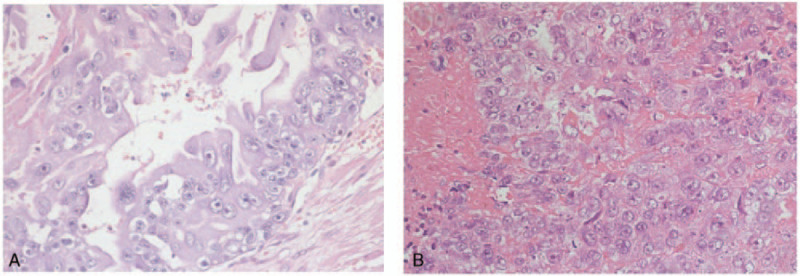
Histopathology of choriocarcinoma in the fallopian tube (Fig. 1A) and placenta (Fig. 1B). Both the tumor in the fallopian tube and the intraplacenta tumor contained a mixture of cytotrophoblastic cells with clear cytoplasm, multinucleated syncytiotrophoblastic cells, and blood. Hematoxylin and eosin staining. Magnification, (A) HE 20×; (B) HE 20×.

At 34 weeks of gestation, a 21-year-old primigravida was transferred to the emergency room of our institution for cephalgia, reduced fetal movements, and left atrial intracavitary thrombus. She had a history of exploratory laparotomy and left salpingectomy at 14^−1^ weeks of the current pregnancy due to tubal rupture and hemorrhagic shock at a county hospital. Pathological assessment had revealed tubal choriocarcinoma (Fig. 1A). At that time, either termination of pregnancy or chemotherapy administration during pregnancy was recommended. She had declined both options. At the subsequent prenatal visits, she underwent only routine prenatal tests and refused all imaging examinations. Her pregnancy was subsequently uneventful, and routine antenatal investigations were normal. Two days prior to her admission at our institution, cephalgia and reduced fetal movements occurred. She was admitted to the county hospital, and echocardiography revealed a left intracavitary thrombus. She was then transferred to our institution and admitted to the intensive care unit. Transthoracic echocardiography showed a 5.2-cm thrombus in the left atrium adjacent to the pulmonary vein that extended through the mitral valve into the left ventricle. Prenatal ultrasound showed a live fetus with decreased amniotic fluid and an amniotic fluid index of 1.26 cm. Diagnoses of left heart thrombus, fetal distress, and oligohydramnios were made. The obstetrician decided to perform a cesarean section to ensure immediate safety of the mother and the fetus. Hence, an emergency cesarean section was performed within 3 hours, and a 1.59 kg live female was born with APGAR scores of 6, 8, and 9 at 1 minute, 5 minutes, and 10 minutes, respectively. Intraoperative exploration showed that the uterus, right fallopian tubes, and both ovaries were negative. Gross examination of the placenta showed that it was approximately 15 × 14 cm in size, with multiple infarcts. Histopathology suggested choriocarcinoma of the placenta (Fig. 1B).

**Figure 2:**
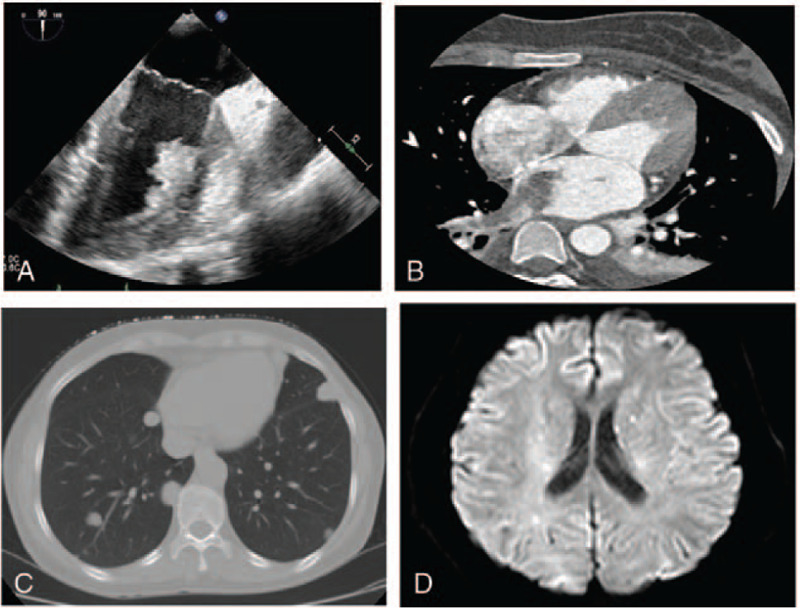
Echocardiogram and pulmonary angiography of Patient. Transesophageal echocardiography (Fig. 2A) and pulmonary angiography revealed a thrombus (Fig. 2B) in the right inferior pulmonary vein contiguous to left atrium and ventriculus. Computed tomography scan of the chest revealed numerous small metastatic lesions (Fig. 3C). Magnetic resonance imaging of the head revealed multiple infarcts and hemorrhages in the brain (Fig. 3D).

**Figure 3:**
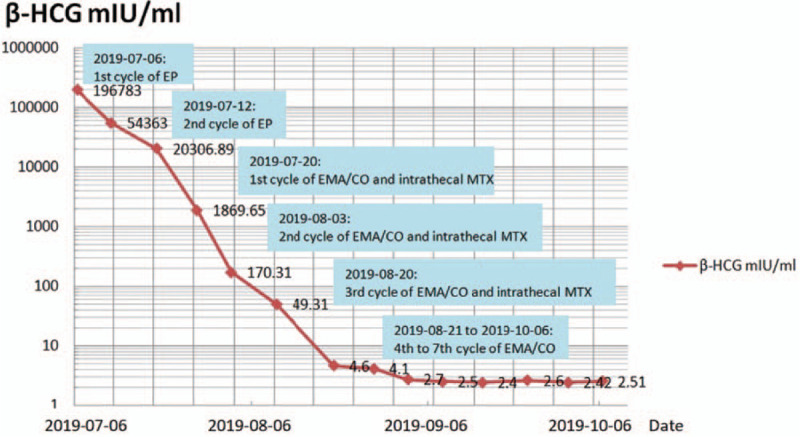
Timeline of serum hCG levels and intervention. Serum hCG levels (IU/L). EP = chemotherapy with etoposide and cisplatin, MTX = methotrexate, EMA/CO = chemotherapy with etoposide, methotrexate, and actinomycin D/cyclophosphamide and vincristine.

After surgery, further investigations were performed. Serum β-human chorionic gonadotrophin(β-HCG) levels were measured, that were found to be highly increased at 196783 IU/l (the normal β-HCG level is <2.9 IU/L). Transesophageal echocardiography revealed a thrombus in the right inferior pulmonary vein contiguous with the left atrium and ventricle (Fig. 2A). Chest computed tomography (CT) and angiography confirmed PVT (Fig. 2B). A chest CT scan confirmed bilateral pulmonary metastases (Fig. 2C). Brain MRI showed multiple cerebral infarcts and hemorrhages (Fig. 2D). Vascular ultrasound indicated thrombosis of the left external iliac artery, right popliteal artery, and right dorsalis pedis artery. Based on these findings, a diagnosis of FIGO stage IV choriocarcinoma with a WHO score of 12 was made. She was therapeutically anticoagulated with low-molecular-weight heparin to mitigate the risk of systemic emboli. In view of the poor condition of the patient, we planned to administer etoposide-cisplatin (EP)- as induced chemotherapy and then initiate etoposide, methotrexate, and actinomycin-D, alternating with cyclophosphamide and vincristine chemotherapy when her general condition improved. On postoperative day 4, chemotherapy with EP was initiated. On the second day after the first EP chemotherapy, the patient's condition worsened, and she presented with symptoms of left limb weakness, slurred speech, and drowsiness. These symptoms worsened over the next few days. Symptomatic treatment and enteral nutrition were provided, which resulted in progressive improvement. These symptoms disappeared after the second cycle of EP chemotherapy (on postpartum day 14). The patient was transferred to the gynecological oncology department. On postpartum day 18, she was administered etoposide, methotrexate, and actinomycin-D, alternating with cyclophosphamide and vincristine chemotherapy. Her cerebrospinal fluid β-HCG value was 3.86 mIU/mL. Three courses of intrathecal methotrexate were also administered treatment of the central nervous system. The patient responded well to chemotherapy. The serum β-HCG levels of the patient became negative after 5 cycles of chemotherapy (Fig. 3). The consolidation therapy comprised 3 additional cycles. Three months after the diagnosis, repeat-echocardiography demonstrated thrombus regression, and repeat-computed tomography scan of the chest was normal, apart from some small residual scars in the location of the previous metastases. The patient was then discharged on rivaroxaban and instructed to follow up with cardiology, pulmonology, and oncology specialists as an outpatient. At the 11-month follow up, the patient remained asymptomatic without complications, and her child was disease-free. Written, informed consent was obtained from the patient for the publication of this report and its accompanying images.

## 3 Discussion

Choriocarcinomas occur infrequently during pregnancy. Although uncommon, choriocarcinoma during pregnancy can be a serious health threat to both the mother and fetus.^[7]^ In this case report, the patient was diagnosed with choriocarcinoma at 14^−1^ weeks of gestation. Chemotherapy is necessary based on the maternal disease status; however, the management of cancer during pregnancy is a clinical dilemma because it involves 2 lives, that of the mother and the fetus. Awareness regarding the fact that chemotherapy can be used during pregnancy is growing. Most standard chemotherapy regimens can be administered between 15 and 35 weeks of gestation.^[8]^ Children exposed to chemotherapy in the second and third trimesters are at risk of preterm delivery, growth restriction, low-birth weight, intrauterine fetal demise, and myelosuppression^[9]^; despite these complications, it is still comparatively safe. However, it should be noted that the optimal therapeutic management of patients with cancer during pregnancy is mainly based on the findings of small, retrospective studies. The most clinical experience in this regard has been obtained for breast cancer, followed by ovarian cancer, while that for gestational choriocarcinoma is minimal. To the best of our knowledge, in the medical literature, only 1 patient with gestational choriocarcinoma received chemotherapy during pregnancy. The patient, who was treated with methotrexate at 22 weeks of gestation, went into spontaneous labor and delivered a live female infant at 25 weeks of gestation.^[10]^ In the current report, the choice of either termination of pregnancy or chemotherapy administration during pregnancy was offered to the patient. This decision was extremely difficult for the mother. Finally, the patient chose to delay chemotherapy until after delivery. Pregnancies with cancer are high risk. Therefore, intensive medical assessment and care are needed both during pregnancy and at delivery. For pregnant women with cancer, planned imaging of the malignancy during pregnancy is necessary.^[4]^ In addition, chemotherapy during pregnancy carries risks that depend primarily on the chemotherapeutics used. Hence, the treatment of patients with cancer during pregnancy should be individualized and performed in specialized centers with considerable experience. Therefore, a multidisciplinary approach and improved education of providers regarding imaging examination and chemotherapy during pregnancy are needed to fully inform patients about treatment options. Moreover, there is a strong need for further research to determine the safety of chemotherapeutic treatments that are routinely used for nonpregnant women, in pregnant women.

In this case report, in addition to gestational choriocarcinoma, the other therapeutic challenge was PVT—a rare but potentially serious and life threatening condition. It is generally thought to be a rare complication of certain primary or secondary lung tumors of the lungs.^[11–13]^ PVT secondary to malignancy is usually a pulmonary vein tumor-thrombus in the pulmonary vein.^[14]^ Without proper and prompt treatment, serious complications including systemic embolization and sudden cardiac death can occur. However, there are no guidelines or expert consensus regarding the treatment of PVT. The management of PVT is often designed according to the cardiopulmonary status and underlying etiology. Treatment of PVT can include anticoagulation, thrombectomy, pulmonary resection, and/or anticancer therapy.^[11]^ In this case report, the patient was diagnosed with choriocarcinoma with PVT and systemic thromboembolism. PVT has rarely been mentioned as a component of choriocarcinoma. In 1966, Maclowry et al reported the first case of choriocarcinoma metastasis extending into the left atrium via the pulmonary vein.^[15]^ To date, only 7 cases of choriocarcinoma complicated by PVT have been reported in the literature. In these cases, PVT is almost always accompanied by diffuse multiorgan metastases such as pulmonary, central nervous system, genital system, and digestive system metastases.^[15–19]^ Prior to the 1990s, there was no effective combination chemotherapy for metastatic choriocarcinoma, and all patients died during diagnostic procedures or soon after the operation, with a mean survival of 3.2 months. Central nervous system hemorrhage was the main cause of death in these patients.^[15,17,20]^ With the advent of effective chemotherapeutic regimens such as EMA-CO and EMA-EP, the survival time of patients with choriocarcinoma with cardiac involvement has been prolonged.^[16,19]^ In this case report, the PVT disappeared spontaneously after chemotherapy combined with anticoagulant therapy. At the 11-month follow-up, the patient remained asymptomatic and had no complications. Our case suggests that conservative therapy can be the first choice for small, asymptomatic PVT secondary to choriocarcinoma. However, it should be noted that patients with PVT are at risk of potentially life-threatening complications directly related to their PVT or as a consequence of treatment. The combination of invasive surgery and chemotherapy is indicated to control acute complications of PVT and to improve the response to chemotherapy protocols.^[11]^ Clinicians should individualize the treatment approach to the patient's specific needs.

## 4 Conclusion

Choriocarcinoma in pregnancy can be a serious health threat to both the mother and the fetus. Management of choriocarcinoma during pregnancy is a clinical dilemma. Therefore, we recommend involving specialized centers with great substantial experience in the management of these patients. In addition, PVT secondary to choriocarcinoma continues to be a therapeutic challenge. For patients with stable cardiopulmonary function, chemotherapy coupled with anticoagulant therapy may be an attractive alternative to urgent surgery.

## Author contributions

**Supervision:** Xiaodong Li.

**Writing – original draft:** Hongfa Peng.
